# In vitro growth inhibitory activity of Medicines for Malaria Venture pathogen box compounds against *Leishmania aethiopica*

**DOI:** 10.1186/s40360-021-00538-2

**Published:** 2021-11-16

**Authors:** Markos Tadele, Solomon M. Abay, Peter Asaga, Eyasu Makonnen, Asrat Hailu

**Affiliations:** 1 Animal Health Research Program, Ethiopian Institute of Agricultural Research, Holetta, Ethiopia; 2 Department of Pharmacology and Clinical Pharmacy, College of Health Sciences, Addis Ababa University, Addis Ababa, Ethiopia; 3 Institute of Human Virology, University Freiburg Medical Centre, Freiburg, Germany; 4 Center for Innovative Drug Development and Therapeutic Trials for Africa (CDT Africa), College of Health Sciences, Addis Ababa University, Addis Ababa, Ethiopia; 5 Department of Microbiology, Immunology and Parasitology, College of Health Sciences, Addis Ababa University, Addis Ababa, Ethiopia

**Keywords:** *Leishmania aethiopica*, Cutaneous leishmaniasis, Pathogen box

## Abstract

**Introduction:**

*Leishmania aethiopica* (*L. aethiopica*) is responsible for different forms of cutaneous leishmaniasis (CL) in Ethiopia. Treatment heavily depends on limited drugs, together with drawbacks like toxicity and microbial resistance. The current research aimed to investigate in vitro growth inhibitory activity of Medicines for Malaria Ventures - Pathogen Box (MMV - PB) compounds against *L. aethiopica* clinical isolate.

**Methodology:**

Four hundred MMV – PB compounds were screened against *L. aethiopica* using resazurin based colourimetric assay. Compounds with > 70% inhibition were further tested using macrophage based intracellular amastigote assay. Cytotoxic and hemolytic activity of candidate hits were assessed on THP1- cells and sheep red blood cells (RBCs), respectively. In vitro drug interaction study was also conducted for the most potent hit using the combination index method.

**Results:**

At the test concentration of 1 μM, twenty-three compounds showed > 50% inhibition of promastigotes parasite growth, of which 11 compounds showed > 70% inhibition. The 50% growth inhibition (IC_50_) of the 11 compounds was ranged from 0.024 to 0.483 μM in anti-promastigote assay and from 0.064 to 0.899 μM in intracellular amastigote assay. Candidate compounds demonstrated good safety on sheep RBCs and THP-1 cell lines. MMV688415 demonstrated a slight hemolytic activity on sheep RBC (5.3% at 25 μM) and THP-1 cell line (CC_20_ = 25 μM) while MMV690102 inhibited half of THP-1 cells at 36.5 μM (selectivity index = 478). No synergistic activity was observed from the combinations of MMV690102 and amphotericin B (CI > 1), and MMV690102 and Pentamidine (CI > 1) at lower and higher combination points.

**Conclusion:**

The present study identified a panel of compounds that can be used as a novel starting point for lead optimization. MMV690102 appears to be the most potent inhibitor against *L. aethiopica* promastigotes and amastigotes. Future works should investigate the antileishmanial mechanism of action and in vivo antileishmanial activities of identified hits.

**Supplementary Information:**

The online version contains supplementary material available at 10.1186/s40360-021-00538-2.

## Introduction

Leishmaniasis is a parasitic disease caused by an obligate intracellular protozoan of the genus *Leishmania.* It is clinically manifested by localized cutaneous leishmaniasis (LCL), diffuse cutaneous leishmaniasis (DCL), mucocutaneous leishmaniasis (MCL) and visceral leishmaniasis (VL) [1, 2]. While VL is typically caused by *Leishmania donovani* and *L. infantum* [3], CL is caused by a broad range of parasites. In the New World, CL is mainly caused by *L. mexicana, L. venezuelensis, L. amazonensis, L. braziliensis, L. panamensis, L. guyanensis* and *L. peruviana*; whereas in the Old World, it is usually caused by *L. major, L. tropica, and L. aethiopica* [4].

Though there are no definite epidemiological data showing the annual burden of CL in Ethiopia, it is estimated to be around 20–50,000 cases/year [5, 6]. In recent times, epidemics of CL and VL were observed in Amhara, Southern Peoples Nations and Nationalities Peoples Regional State and Tigray regions. The number of new cases for CL in these areas was estimated to be 10–50 per 10,000 people [7].

Ethiopian CL is almost always caused by *L. aethiopica* and rarely by *L. tropica* and *L. major. Leishmania aethiopica* is rarely encountered outside endemic area, it is predominantly restricted to Ethiopia, Kenya and Uganda [8] as well as Eritrea [9]. This parasite produces several interesting and peculiar clinical presentations. LCL is the most common form and most at times, it is self-healed while the DCL, and MCL forms of the disease have no tendency of self-cure [6] and requires systemic antileishmanial treatments. The disease is transmitted mainly by two species of phlebotomine (*Phlebotomus pedifer and P. longipes*) sand flies although *L. aethiopica* and *L. tropica* were recovered from *P. sergenti* and *P. saevus,* respectively [10].

Treatment for LCL is only recommended when lesions are mucosal, diffused, and complicated and if patients are immunocompromised. Current treatment trends are based on chemotherapy with systemic use of antileishmanial drugs like sodium stibogluconate, pentamidine, amphotericin B, paromomycin and miltefosine [6, 11]. Physical therapies with liquid nitrogen (Cryotherapy) [12], heat therapy for treating lesions at 50 °C for 30 s up to three times are also practised [1].

Existing drugs show variable treatment responses in different parts of the country. Teklemariam et al. (1994) [13], Fikre et al. (2017) [14] and Zanger et al. (2011) [9] reported mixed therapeutic responses in DCL, MCL and LCL patients treated with common antileishmanial drugs. These reports noted very poor responses and the need for extended (up to 3.5 months) treatment duration for DCL and MCL. There is therefore the need for alternative therapeutics that can be used in combination with currently used antileishmanial drugs for the treatment of Ethiopian CL*.*

The PB contains 400 pure compounds active against various neglected tropical diseases. It has been previously screened for *Trypanosoma brucei* [15], *Giardia lamblia* [16] *Cryptosporidium parvum* and *Plasmodium falciparum* [17], *Toxoplasma gondii* [18], and recently against *L. donovani* [19] to uncover new starting points for antiprotozoal drug discovery. In this study, we screened the MMV PB to expand the pool of antileishmanial drugs active against clinical *L. aethiopica* promastigote and intracellular amastigote in vitro. This report is an independent screening of the PB against *L. aethiopica*.

## Materials and methods

### Preparation of test and reference compounds

The PB, composed of 400 compounds, was supplied in sealed plates containing a frozen 10 μL of 10 mM dimethyl sulfoxide (DMSO), solutions in 96-well plates. The original master plates were copied into 10 plates containing 10 μL solutions at 1 mM concentration and stored at − 20 °C till used. Amphotericin B, pentamidine isothionate, miltefosine (all from Sigma-Aldrich Che. Co., St. Louis, USA) were used as reference drugs (positive control). All compounds including the standard reference drugs were diluted with DMSO (Sigma Aldrich, Co., USA), intermediate and final dilutions were made by M199, RPMI and MEM complete media according to the assay type.

### Test strain, cell line, laboratory animals

*Leishmania aethiopica* isolate (579/17) was obtained from Leishmaniasis Research and Diagnostic Laboratory (LRDL), Addis Ababa University. The parasite was isolated from 50-year-old female patient residing in Ambo, Western Shoa zone, Oromia region, Ethiopia in 2017. The patient had no history of CL infection and was not treated with any kind of antileishmanial drugs before the diagnosis. The isolate was previously characterized according to the method described by [20]. Both the reference strains and the isolate were treated with standard antileishmanial drugs to see any drug susceptibility differences between them. THP-1 cell lines were obtained from Armauer Hansen Research Institute (AHRI), Addis Ababa. Swiss albino mice were obtained from college of health sciences, Addis Ababa University animal house. All procedures and techniques used in this study were in accordance with the national institute of health guidelines for the care and use of laboratory animals (Institute for Laboratory Animal Research, 2011) and following the ARRIVE Guidelines Checklist.

### Culture conditions

#### Leishmania parasite isolation and culture

promastigotes were isolated using NNN media. Logarithmic stage *L. aethiopica* promastigotes were transferred to tissue culture flasks containing medium 199 (M199) supplemented with 15% heat inactivated new born calf serum (HINBCS), 25 mM 4-(2-hydroxyethyl)-1-piperazineethanesulfonic acid (HEPES), 2 mM L-glutamine, 100 IU/mL penicillin and 100 μg/mL streptomycin solution (all from Sigma-Aldrich, Co., St. Louis, USA) and incubated at 22 °C in Plate Shaker Incubator (Gerhardt).

#### THP-1 cell line cultures

THP-1 cells were cultured in RPMI 1640 Medium (Sigma-Aldrich, Co., St. Louis, USA) supplemented with 10% HINBCS, 100 IU/mL penicillin, 100 μg/mL streptomycin at 37 °C in 5% CO_2_ humidified incubator (Thermo Electron).

#### Intra-peritoneal macrophage collection and culture

Macrophages were collected from Swiss albino mice as described briefly in our previous work [19]. Young mice (6–8 weeks old) were injected into the peritoneal cavity with 2% freshly prepared starch (Sigma-Aldrich, Co., St. Louis, USA) and 10 mL of sterile ice-cold phosphate-buffered saline (PBS) (Sigma, Co., St. Louis, USA) supplemented with 3% HINBCS after 2 days, and 6–8 mL exudates were recovered. The Contents were centrifuged at 450 g for 10 min and macrophages were resuspended in essential medium (MEM) (Sigma-Aldrich, Co., St. Louis, USA) containing 10% HINBCS, 25 mM HEPES, 2 mM L-glutamine and 100 IU/mL penicillin and 100 μg/mL streptomycin.

### Biological assay

#### Primary antipromastigote assay

Test compounds were diluted in 96-well microculture plate containing 100 μL complete M199 medium. Parasite suspension (1 × 10^6^ promastigotes/mL) in 100 μL media volume was then added to each well to achieve a final concentration of 1 μM. Plates were incubated for 72 h at 22 °C in the Plate Shaker Incubator and cultured with blank wells, as well as positive (amphotericin B, miltefosine, pentamidine) and negative (DMSO at < 1% v/v concentration) controls.

The IC_50_ of test compounds against promastigote stages were performed in triplicates in a twofold dose-titration range (0.03 to 1 μM). The Parasite density was kept constant at 1 × 10^5^ per well. After 68 h of incubation, 10 μL of fluorochrome resazurin solution (Sigma-Aldrich, Co., St. Louis, USA) was added and the reduced resazurin was measured after an incubation time of 72 h at 530 nm excitation wavelength and 590 nm emission wavelength. The percentage of inhibition (%) was calculated for each concentration using the following formula:

$$ \mathrm{Inhibition}\ \left(\%\right)=100-\frac{\ \left(\mathrm{Fluorescence}\ \mathrm{in}\ \mathrm{duplicate}\ \mathrm{drug}\ \mathrm{wells}-\mathrm{Fluorescence}\ \mathrm{in}\ \mathrm{blank}\ \mathrm{wells}\right)}{\left(\mathrm{Fluorescence}\ \mathrm{control}\ \mathrm{wells}-\mathrm{Fluorescence}\ \mathrm{in}\ \mathrm{blank}\ \mathrm{wells}\right)}\ast 100 $$

#### Haemolysis test

Serially titrated (1.56–25 μM) test compounds were mixed with 2% blood suspension in Eppendorf tubes and incubated at 37 °C for two hours with Triton X-114 (5 μL/mL) (Sigma-Aldrich, Co., St. Louis, USA) and 2.5% DMSO as positive and negative controls. The mixture was then centrifuged at 1000 g for 10 min. 75 μL of the resulting supernatant were transferred to 96-well plates and the absorbance readings obtained at 540 nm using Victor3 Multilabel Counter (PerkinElmer, Waltham, USA).

#### THP-1 cell cytotoxicity assay

Approximately 2 × 10^5^ THP-1 cell suspension were added to 96-well microplate containing serially diluted 50 μL test compounds*.* Microplates were then incubated for 68 h at 37 °C in a 5% CO_2_ air mixture. Cell viability was determined with the resazurin- based fluorescence assay measured after a total incubation time of 72 h. The estimated median cell cytotoxicity (CC_50_) was calculated for each concentration using the formula:

$$ \mathrm{Cell}\ \mathrm{viability}\ \left(\%\right)=\frac{\left(\mathrm{Fluorescence}\ \mathrm{in}\ \mathrm{duplicate}\ \mathrm{drug}\ \mathrm{wells}-\mathrm{Fluorescence}\ \mathrm{blank}\ \mathrm{wells}\right)}{\left(\mathrm{Fluorescence}\ \mathrm{in}\ \mathrm{control}\ \mathrm{wells}-\mathrm{Fluorescence}\ \mathrm{blank}\ \mathrm{wells}\right)}\ast 100 $$

#### Selectivity index (SI)

The selectivity index of compounds was calculated from the ratio of the CC_50_ value determined in THP1 cells to the IC_50_ value determined from the dose-response curves in promastigote and amastigote assay using the following formula.

$$ \mathrm{Selectivity}\ \mathrm{index}\ \left(\mathrm{SI}\right)=\frac{{\mathrm{CC}}_{50}\ \mathrm{THP}1\ \mathrm{cells}}{{\mathrm{IC}}_{50}\ \mathrm{promastigotes}\ \mathrm{or}\ \mathrm{amstigotes}\ } $$

#### Physicochemical properties

Physicochemical properties of the compounds were obtained from National Centre for Biotechnology Information (NCBI) (www.ncbi.nlm.nih.gov). The obtained physicochemical data was then checked for compliance with Lipinski’s rule of absorption and permeation [21].

#### Intracellular amastigote assay

Intracellular amastigote assay was conducted according to the method described elsewhere [19, 22] with modifications. Approximately 3 × 10^5^ peritoneal macrophages were seeded in 24-well plates and allowed to adhere for 12 h at 33 °C in 5% CO_2_. Adherent cells were infected with late-stage *L. aethiopica* promastigotes (1:10 ratio) after washing out non-adherent cells twice. Only when > 70% of the macrophages present in the wells were infected was the infection deemed sufficient. The medium was then replaced with a fresh complete medium with or without test compounds and incubated for 72 h at 33 °C and 5% CO_2_. After 72 h of incubation, slides were washed with prewarmed PBS, fixed with methanol, and stained with Giemsa (10%) for 15 min and observed under 100x oil immersion objective microscope [Olympus]. Amphotericin B and pentamidine were used as reference drugs against test samples.

#### Determination of IC_50_ against intracellular amastigotes

The IC_50_ was determined by counting amastigotes in (50) macrophages in duplicate cultures. In all assays except for haemolytic and THP- 1**,** test compounds were diluted in a t**wo**-fold dose-titration range (0.03 to 1 μM) in triplicate. Parasite burden was calculated using the infection index as shown below.

$$ \mathrm{Infection}\ \mathrm{in}\mathrm{dex}=\frac{\mathrm{Number}\ \mathrm{of}\ \mathrm{in}\mathrm{fected}\ \mathrm{macrophage}\mathrm{s}}{\ \mathrm{Total}\ \mathrm{macrophage}\ \mathrm{counted}}\mathrm{x}\frac{\ \mathrm{Total}\ \mathrm{number}\ \mathrm{of}\ \mathrm{amastigotes}\ \mathrm{in}\ \mathrm{in}\mathrm{fected}\ \mathrm{macrophage}\mathrm{s}}{\mathrm{Total}\ \mathrm{in}\mathrm{fected}\ \mathrm{macrophage}\mathrm{s}\ \mathrm{counted}} $$

The IC_50_ of each test compound was defined as the inhibitory concentration of test compounds that reduces the number of amastigotes per infected macrophages by 50%.

#### Evaluation of the synergistic activity of molecules

In vitro drug interactions were assessed using the combination index method as described by Chou and Martin [23]. The dose-response relationship of each drug alone and in combination was assessed separately as illustrated in Table 1.

**Table 1:** Layout of a combination experiment for synergistic effect of two drugs

Drug 1	4(IC_50_) = A	2(IC_50_) = C	**(IC**_**50**_**) = E**	(IC_50_)/2 = G	(IC_50_)/4 = I
Drug 2	4(IC_50_) = B	2(IC_50_) = D	**(IC**_**50**_**) = F**	(IC_50_)/2 = H	(IC_50_)/4 = J
**Combination**	**A + B**	**C + D**	**E + F**	**G + H**	**I + J**

The combination index (CI) values were estimated to be CI < 1, =1, and > 1 which indicate synergism, additive, and antagonism, respectively

### Statistical analysis

Percentage inhibition for each test and reference compounds were expressed as mean values ±95% confidence interval (CI). The IC_50_ values were determined by GraphPad Prism version 8.4 (GraphPad software, inc. 2020) using non-linear regression model: Y = 100/ (1 + 10^((LogIC_50_ - X)*Hillslope))). Where, X = log of concentration, Y = Normalized response (0–100%), logIC_50_ = same log units as X HillSlope = Slope factor or Hill slope. Pooled data were expressed as means ±95% CI of two independent experiments, with each test concentration in triplicates. Drug combination effects of each selected compound with their references were assessed using isobologram and the analysis was made using Compusyn (CompuSyn 1.0, ComboSyn, Inc., 2005).

## Results

### Primary screening and dose titration assay

**Fig. 1:**
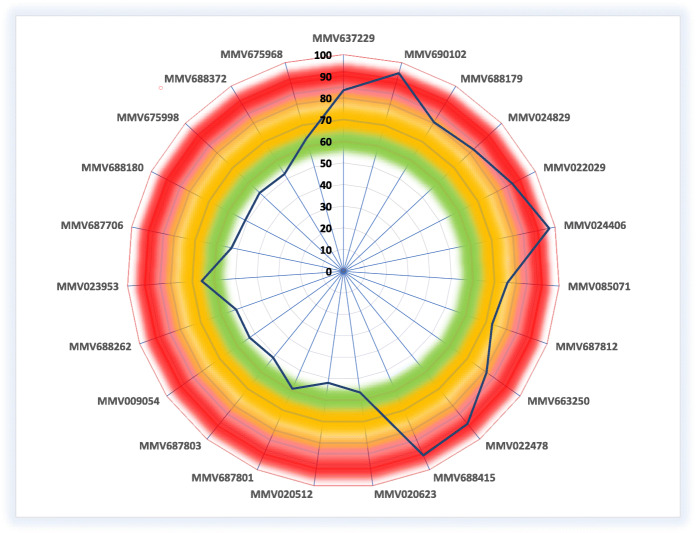
Radar graph illustrating the percent inhibition of PB compounds against *L* aethiopica promastigotes at 1 μM. The red colour corresponds to the highest inhibitions (more than 85%). The orange zone represents 70–85% inhibition, while the green zone corresponds to 50–70% inhibition. The experiment was conducted using amphotericin B and Pentamidine as standard

The preliminary screening uses *L. aethiopica* promastigotes. All 400 compounds were screened at 1 μM (Supportive file Fig. 1 and Supportive file Table 1)*.* From which, 23 compounds had > 50% inhibitory effect in the initial screening process. Eleven of the 23 compounds had an inhibitory activity of > 70% as shown in the radar map (Fig. 1). The radar was developed using the average percentage of each inhibitor compound obtained from two independent experiments conducted in triplicates.

Active compounds which demonstrated > 70% promastigote inhibitory activity were selected for further analysis. The IC_50_ values for all tested compounds were below 0.483 μM; of these, MMV024406, MMV688415 and MMV690102 demonstrated IC50 of 0.024 μM, 0.145 μM and 0.158 μM, respectively on the primary dose titration assay performed on promastigotes (Table 2).

**Table 2:** Activity of Selected Compounds against *Leishmania aethiopica* promastigotes

Plate–well location	MMV ID	IC_50_ (95% CI)	Cytotoxicity, CC_50_ (SI), μM		Mode of action	Previous activity report
			Sheep RBC	THP-1		
B-F5	MMV637229	0.483 (0.340–0.740)	> 25 (**ND**)	> 25 (**ND**)	Antihistamine	*T. cruzi*, *L. donovani* [19, 24]
C-E8	MMV690102	0.158 (0.086–0.213)	> 25 (**ND**)	36 (478) *	DHFR inhibitors	*L.donovani*, *L.mexicana* [24, 25]
C-F3	MMV688179	0.356 (0.180–0.440)	> 25 (**ND**)	> 25 (**ND**)	DNA interfere with DNA multiplication	*T. brucei* and *L. donovani* [24]
D-B5	MMV024829	0.356 (0.174–0.352)	> 25 (**ND**)	> 25 (**ND**)	–	–
D-B7	MMV022029	0.305 (0.241–0.364)	> 25 (**ND**)	> 25 (**ND**)	–	*T. brucei* [24]
D-D11	MMV024406	0.024 (0.016–0.031)	> 25 (**ND**)	> 25 (**ND**)	–	*C. albicans* [26]
D-E4	MMV085071	0.283 (0.110–0.471)	> 25 (**ND**)	> 25 (**ND**)	DV disruption, mitochondrial degradation & DNA fragmentation	*P. falciparum* [27]
D-F11	MMV687812	0.390 (0.285–0.502)	> 25 (**ND**)	> 25 (**ND**)	DV-disrupting/ DNA degradation	*P. falciparum* [27]
D-G6	MMV663250	0.326 (0.190–0.530)	> 25 (**ND**)	> 25 (**ND**)	–	–
D-H3	MMV022478	0.448 (0.138–0.580)	> 25 (**ND**)	> 25 (**ND**)	NADPH oxidase inhibitors via inhibition of protein kinase C [28]	*L. donovani, T. gondi* [18, 19, 24]
E-G11	MMV688415	0.145 (0.102–0.180)	> 25 (**ND**)	> 25 (**ND**)	–	*T. cruzi* [24]
Amphotericin B		0. 106 (0.06–0.140)				
Pentamidine		1.31 (0.728–1.92)				

Description: *95% CI* 95% confidence interval, *IC*_*50*_ Median inhibitory concentration, *DV* digestive vacuole, *SI* selectivity index, *ND* not determined, ^*^extrapolated result. The IC_50_ value indicated was the average of two independent experiments conducted in triplicate

**Fig. 2:**
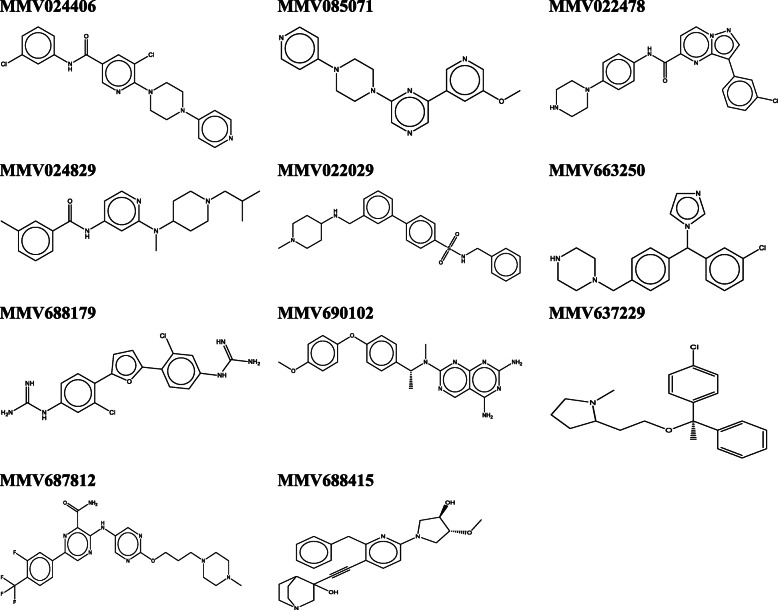
Structures of pathogen box compounds with > 70% promastigote growth inhibition at 1 μM

The molecular structure of the most potent (> 70% inhibition) Pathogen Box compounds against *L. aethiopica* promastigotes are indicated in Fig. 2.

### Cytotoxicity study

The study shows MMV688415 to have hemolytic activity on sheep RBC (5.3% hemolytic activity at 25 μM) and THP-1 cell line (CC_20_ = 25 μM). MMV690102 it also demonstrated an inhibitory activity on THP-1 cell lines with an extrapolated IC_50_ value of 36.55 μM (95% CI: 29.86–45.36). The calculated selectivity index (SI) for MMV690102 was 478.4.

### Physicochemical properties

MMV687812 was found to be higher than the 500 g/mol molecular weight and the 10-hydrogen bond receiver count. This clearly violates Lipinski’s rule of permeation and absorption. Hence, it was discarded from further screening (Table 3).

**Table 3:** Physicochemical properties of selected compounds

MMV ID	R1	R2	R3	R4	R5	R6	Reference
MMV688179	403.3	4	3	4	2.5	142	[29]
MMV085071	348.4	0	7	4	1.3	67.3	[30]
MMV688415	433.5	2	6	6	2.3	69.1	[31]
MMV024406	428.3	1	5	4	3.9	61.4	[32]
MMV663250	366.1	1	3	5	3.1	33.1	[33]
MMV637229	343.9	0	2	6	5	12.5	[34]
MMV690102	417.4	2	9	6	3.3	125	[35]
MMV022478	432.9	2	5	4	3.2	74.6	[36]
MMV024829	380.5	1	4	6	4.4	48.5	[37]
MMV687812	534.5	2	13	9	2.9	122	[38]
MMV022029	449.6	2	5	8	4	69.8	[39]

Description: *R1* Molecular weight (g/mol), *R2* H^+^ bond donor, *R3* H^+^ bond receiver, *R4* Rotatable bonds, *R5* XLogP3-AA, *R6* Polar surface area (A^2^). Lipinski’s rule of five (RO5) associate compounds with more than 5 H-bond donors, 10 H-bond acceptors, molecular weight greater than 500 and computational log P (ClogP) greater than 5 (or Moriguchi log P [MLogP] > 4.15) poor absorption and/or permeation

### Activity of selected compounds against *Leishmania aethiopica* amastigotes

**Fig. 3:**
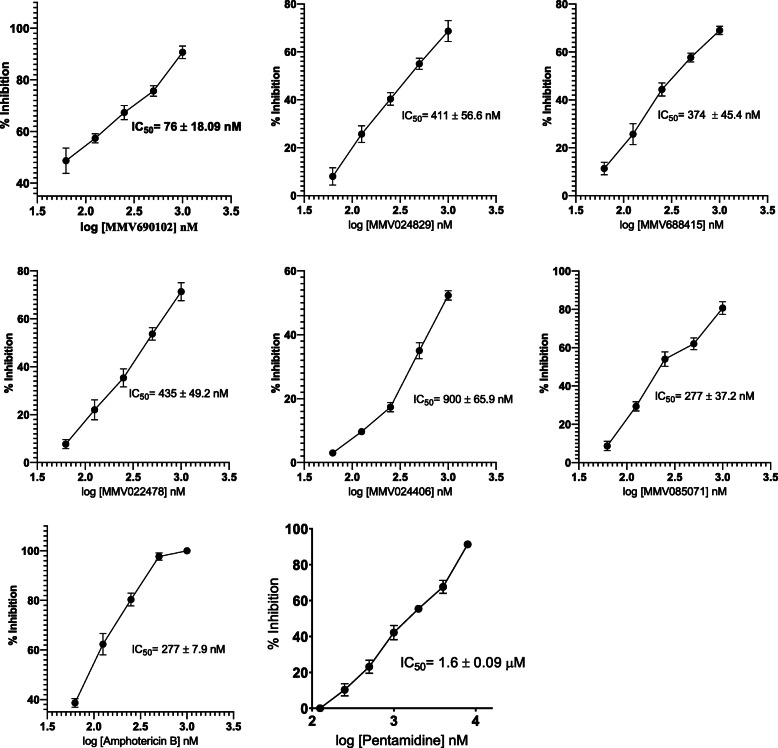
Showing a maximum 50% inhibitory concentration against intracellular *L. aethiopica* amastigotes

Ten compounds were screened at 1 μM for their activity against intracellular amastigotes. Out of these, only six compounds showed > 50% inhibition at 1 μM. The percentage of inhibition observed at 1 μM was MMV690102 (90.7 ± 5.3), MMV085071 (80.5 ± 4.2), MMV688415 (68.6 ± 6.6), MMV022478 (72.4 ± 5.6), MMV024829 (69.6 ± 4.1) and MMV024406 (55.6 ± 5.5). The remaining 4 compounds could not produce > 50% intracellular amastigote reduction. MMV690102 was found to be a very potent inhibitor of *L. aethiopica* amastigotes (Fig. 3). MMV biological data also showed IC_50_ value 3 nM on *L. infantum*, 20-fold lower.

### Synergistic effect of the identified hits with reference drugs

Based on the performance of the intracellular amastigotes assay, MMV690102 was evaluated for its synergistic effect with pentamidine and amphotericin B. The combination index values for the two combination MMV690102-pentamidine (ED_50_ = 2.67, ED_75_ = 2.54, ED_90_ = 2.42, ED_95_ = 2.34) and MMV690102-amphotericin B (ED_50_ = 1.88, ED_75_ = 2.18, ED_90_ = 2.56, ED_95_ = 2.88) were > 2, indicating the combination have no synergistic effect and are less relevant to combination therapy (supportive file Fig. 2).

## Discussion

The sole purpose of the primary screening was to generate a complete hit map for all 400 compounds at 1 μM (Supplemented Fig. 1). The map was developed using the average percentage of inhibition of each compound obtained from two independent experiments in triplicate. The list of compounds that showed > 50% inhibitive activity is presented in the radar graph (Fig. 1). Of which, 11 compounds with > 70% inhibition were taken for further investigation in a dose titration assay against *L. aethiopica* promastigotes.

MMV690102 was found to be a very potent inhibitor of both forms of the parasite. This finding is consistent with the reports of Berry et al. [25] and Duffy et al. [24] which used *L. Mexicana* and *L. donovani* strains, respectively. We previously reported MMV690102 as one of the potent growth inhibitors of *L. donovani* promastigotes and amastigotes [19]. This indicates the potentials of this compound for the treatment of infections caused by more than one *Leishmania* species types. MMV085071, MMV022478 and MMV688415 also exhibited good activities on *L. aethiopica* intracellular amastigotes (Fig. 2).

The present study showed that the calculated SI of MMV690102 based on the cytotoxicity activity against THP-1 cell lines was greater than 100 (SI = 478.4). MMV reported IC_50_ value of 5.4 μM and 4 μM against MRC-5 and PMM cells. Other useful information such as DMPK are also available from the MMV PB website www.mmv.org/mmv-open/pathogen-box/about-pathogen-box#composition. The inhibition observed on these two mammalian cells is lower compared to the effect observed on THP-1 and RBC in this study. It is therefore very imperative to conduct additional cytotoxicity studies on other mammalian cells. Compounds under the same class are less selective between the host cells and other DHFR expressing eukaryotes.

MMV688179 is analogue of furamidine, which is an amphipathic diamine antiprotozoal drug. Though the mechanism of action is not clearly indicated, the analogues are known to bind with DNA at the AT sites and form DNA–drug complexes and interfere with replication [40, 41]. MMV022478 is a member of the pyrazolo [1,5-a] pyrimidine class which has been identified as an inhibitor of NADPH oxidase through inhibition of protein kinase C [28, 42]. The activity of MMV688415 and MMV022478 against *T. cruzi* [24] and *Toxoplasma gondii and T. cruzi* [24, 43]*,* had previously been reported, without compelling evidence on their biological target. MMV637229 had been reported to inhibit histamine receptor [44]. MMV687812 was identified as a potent digestive vacuole (DV) disruptive antimalarial agent, with the most pronounced DV-disrupting DNA degradation capacities [27]. The novelty of this screening is the first of its kind on *L. aethiopica* species, as a result, there was no published work to compare our findings.

MMV022029 and MMV024406 demonstrated high inhibition properties at 1 μM in the promastigote growth inhibition assay. However, their activities against the intracellular amastigotes were low. Reduction of activity in the intracellular assay is expected as these compounds traverse through the membranes to reach their site of action [45, 46]. A significant reduction of activity like this one might suggest poor membrane permeability and stability in the acidic and hydrolytic environment of parasitophorous vacuoles [47] or maybe the biological target variation between promastigotes and amastigotes forms.

Poor membrane permeability is directly associated with XLogP3-AA values. The physiochemical data gathered from NCBI (Table 3) showed MMV022029 and MMV024406 having XLogP3-AA value of 3.9 and 4, respectively, which is close to the cut-off point for computational log P (ClogP) for acceptable absorption or permeation. This might play a role in poor intracellular inhibitory activity as membrane permeability is inversely correlated with XLogP3-AA value. In addition to drug permeation property, inherent differences in transcriptomes and proteosomes of amastigotes and promastigotes may cause difference in susceptibility [48]. The cell growth and metabolism difference between promastigotes and amastigotes can also be a factor. The former unlike the latter is known for its high and rapid proliferation [48] which makes it susceptible for growth-limiting agents.

## Conclusion

In this study, five compounds were identified as confirmed hits agents for *L. aethiopica*. Further In vivo and in vitro analytical assessments are needed to evaluate their safety, pharmacokinetic profile and antileishmanial mechanism using target-based experiments. We performed the intracellular assay based on the activity of compounds in the preliminary assay. This method might mask compounds with low inhibitory activity on promastigotes, otherwise effective against the intracellular stage. We, therefore, recommend high throughput screening of the library against the amastigote forms. Majority of the compounds identified in this study lacked relevant biological data. Therefore, their pharmacokinetics studies, as well as related activities on mammalian cells, should also be conducted.

In the intracellular assay, the performance of MMV022029 and MMV024406 were significantly reduced; structural optimization can be conducted to improve target binding affinity, physicochemical and pharmacokinetic properties, and maximize the in vitro potency. Moreover, in vivo animal models can also be considered to check if there is a need for bioactivation.

## Supplementary Information

**Additional file 1: Supportive file Fig. 1.** Heat map of percent inhibition of pathogen box compounds against Leishmania aethiopica promastigotes.

**Additional file 2: Supportive file Fig. 2.** Dose–effect curve and isobologram analysis for synergistic effect of MMV690102 with amphotericin B [1:1]) and pentamidine combination [1:20].

**Additional file 3: Supportive file Table 1.** Hit map with actual Percentage of inhibition of MMV pathogen Box compounds against *Leishmania aethiopica* promastigotes at 1 μM.

## Data Availability

The datasets supporting the conclusions of this article are included within the article. Additional data will be available upon request from corresponding author.
