# Supplementary material for: In vitro growth inhibitory activity of Medicines for Malaria Venture pathogen box compounds against Leishmania aethiopica
Source: BMC Pharmacol Toxicol. 2021 Nov 16;22:71. doi: 10.1186/s40360-021-00538-2 (PMC8594108; doi:10.1186/s40360-021-00538-2)
Supplement: Supplementary file 1 — Additional file 1: Supportive file Fig. 1. Heat map of percent inhibition of pathogen box compounds against Leishmania aethiopica promastigotes. [file 40360_2021_538_MOESM1_ESM.pdf]

In vitro growth inhibitory activity of Medicines for Malaria Venture Pathogen Box compounds against Leishmania aethiopica clinical isolates

Markos Tadele, Solomon M. Abay, Peter Asaga, Eyasu Makonnen, Asrat Hailu

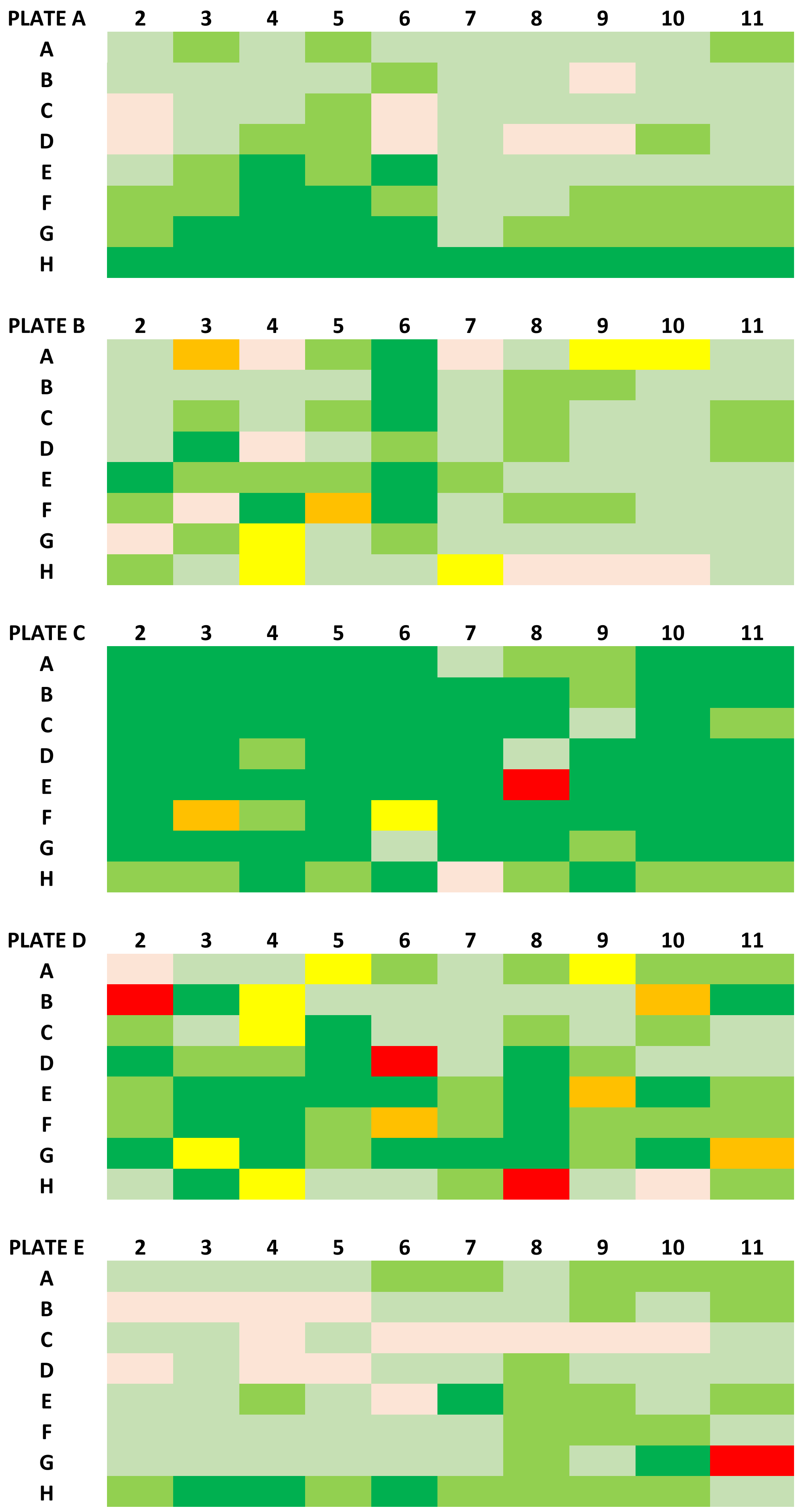

Supported file Fig S01. Heat map of percent inhibition of pathogen box compounds against *Leishmania aethiopica* promastigotes. The red and brown color corresponds to highest inhibitions (more than 70%); Yellow color parallels with 50-69.9% inhibition while light green and dark green colors correspond to lowest inhibition (less than 50%). The experiment was conducted using amphotericin B and Pentamidine as standard drugs. The heat map was generated using the average activity values of compounds from each microplate well in two independent experiments conducted in triplicate.
