# Supplementary material for: In vitro growth inhibitory activity of Medicines for Malaria Venture pathogen box compounds against Leishmania aethiopica
Source: BMC Pharmacol Toxicol. 2021 Nov 16;22:71. doi: 10.1186/s40360-021-00538-2 (PMC8594108; doi:10.1186/s40360-021-00538-2)
Supplement: Supplementary file 2 — Additional file 2: Supportive file Fig. 2. Dose–effect curve and isobologram analysis for synergistic effect of MMV690102 with amphotericin B [1:1]) and pentamidine combination [1:20]. [file 40360_2021_538_MOESM2_ESM.pdf]

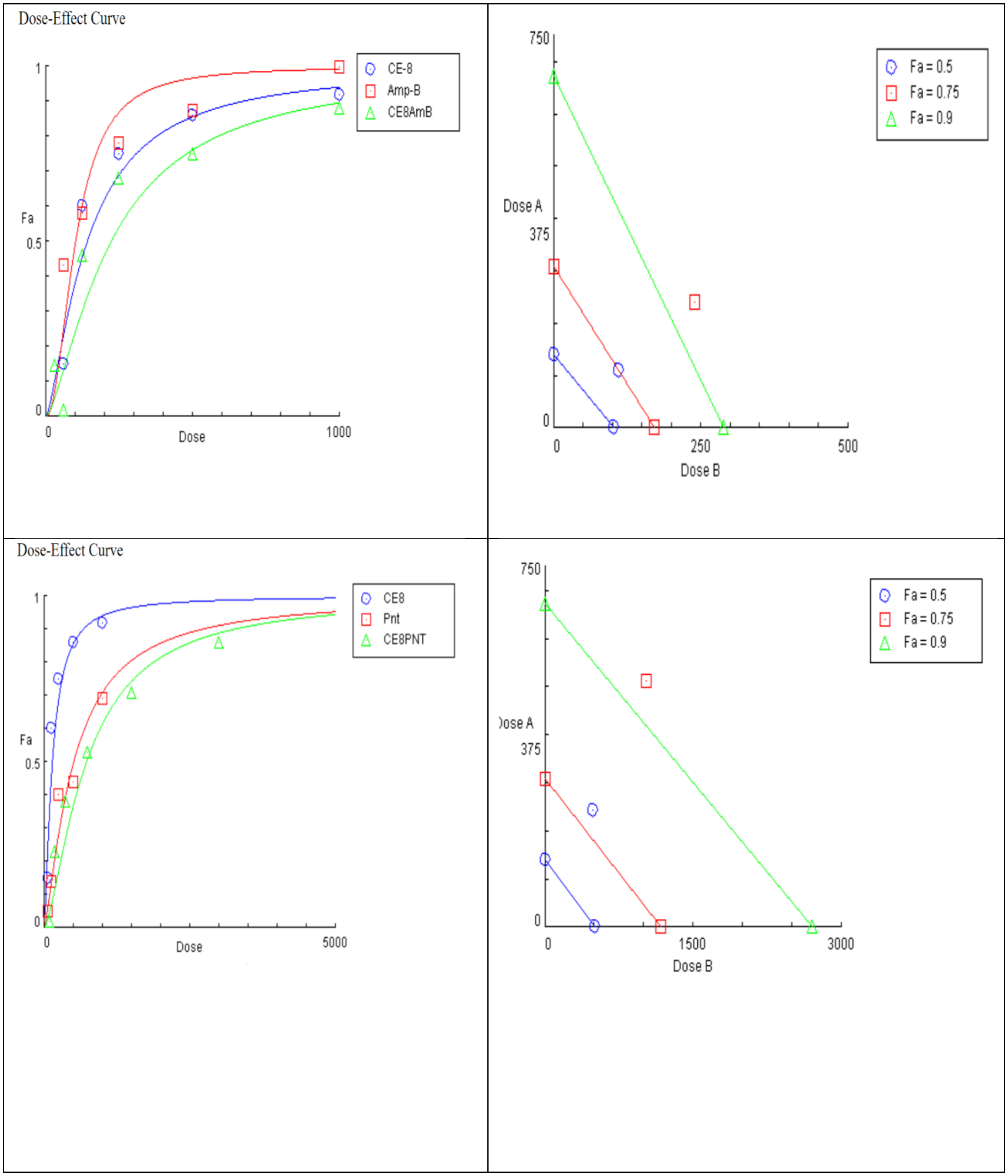

Fig. Dose–effect curve (Left) and isobologram analysis (Right) for synergistic effect of MMV690102 with amphotericin B [1:1]) and pentamidine [1:20] combination.

Drug: MMV690102 (CE8) [nM]
Drug: Pentamidine (Pnt) [nM]
Drug Combo: Combination (CE8+Pnt [1:20])

	CI values at:			
**Combo**	**ED50**	**ED75**	**ED90**	**ED95**
(MMV690102 (CE8) + pentamidine)	2.673	2.544	2.423	2.346

Drug: MMV690102 (CE8) [nM]
Drug: Amphotericine B (Amp-B) [nM]
Drug Combo: Combination for (MMV690102 (CE8) + Amphotericin B) ([1:1])

	CI values at:			
**Combo**	**ED50**	**ED75**	**ED90**	**ED95**
CE8AmB	1.88396	2.17965	2.56059	2.88024
